# Environmental Surveillance of *Vibrio cholerae* O1/O139 in the Five African Great Lakes and Other Major Surface Water Sources in Uganda

**DOI:** 10.3389/fmicb.2018.01560

**Published:** 2018-08-03

**Authors:** Godfrey Bwire, Amanda K. Debes, Christopher G. Orach, Atek Kagirita, Malathi Ram, Henry Komakech, Joseph B. Voeglein, Ambrose W. Buyinza, Tonny Obala, W. Abdullah Brooks, David A. Sack

**Affiliations:** ^1^Department of Community Health, Ministry of Health, Kampala, Uganda; ^2^Department of Quality Control, National Drug Authority, Ministry of Health, Kampala, Uganda; ^3^Department of International Health, Johns Hopkins Bloomberg School of Public Health, Baltimore, MD, United States; ^4^Community and Behavioral Sciences, College of Health Sciences, Makerere University School of Public Health, Kampala, Uganda; ^5^Uganda National Health Laboratory Services – Central Public Health Laboratory, Ministry of Health, Kampala, Uganda; ^6^Department of Geography, Makerere University, Kampala, Uganda

**Keywords:** lakes, *Vibrio cholerae*, reservoir, Uganda, PCR, Africa, environment surveillance, water pathogens

## Abstract

Cholera is a major public health problem in the African Great Lakes basin. Two hypotheses might explain this observation, namely the lakes are reservoirs of toxigenic *Vibrio cholerae* O1 and O139 bacteria, or cholera outbreaks are a result of repeated pathogen introduction from the neighboring communities/countries but the lakes facilitate the introductions. A prospective study was conducted in Uganda between February 2015 and January 2016 in which 28 selected surface water sources were tested for the presence of *V. cholerae* species using cholera rapid test and multiplex polymerase chain reaction. Of 322 water samples tested, 35 (10.8%) were positive for *V. cholerae* non O1/non O139 and two samples tested positive for non-toxigenic atypical *V. cholerae* O139. None of the samples tested had toxigenic *V. cholerae* O1 or O139 that are responsible for cholera epidemics. The Lake Albert region registered the highest number of positive tests for *V. cholerae* non O1/non O139 at 47% (9/19). The peak period for *V. cholerae* non O1/non O139 positive tests was in March–July 2015 which coincided with the first rainy season in Uganda. This study showed that the surface water sources, including the African Great Lakes in Uganda, are less likely to be reservoirs for the observed *V. cholerae* O1 or O139 epidemics, though they are natural habitats for *V. cholerae* non O1/non O139 and atypical non-toxigenic *V. cholerae* O139. Further studies by WGS tests of non-toxigenic atypical *V. cholerae* O139 and physicochemical tests of surface water sources that supports *V. cholerae* should be done to provide more information. Since *V. cholerae* non O1/non O139 may cause other human infections, their continued surveillance is needed to understand their potential pathogenicity.

## Introduction

Cholera, a preventable and treatable infectious disease, is a major contributor to morbidity and mortality in many countries in the world (Mengel et al., 2014; Ali et al., 2015). Cholera is caused by the bacteria *Vibrio cholerae*. There are >200 serotypes of *V. cholerae* but only serotypes O1 and O139 are responsible for cholera epidemics (Cabral, 2010). Cholera appeared in Africa in the early 1970s, and then spread to 30 out of, the then, 46 countries in the continent (Glass et al., 1991). Similarly, in recent years, over 41% of all cholera cases and deaths reported to the World Health Organization (WHO) were from African countries (World Health Organization, 2016b). The top 10 African countries reporting cholera cases and deaths annually to WHO were in sub-Saharan Africa and included Uganda (World Health Organization, 2013, 2015; World, 2016a). The consequences of cholera epidemics may be severe and lead to national crises in some African countries (Mason, 2009; Morof et al., 2013).

Cholera first appeared in Uganda in the early 1970s as small, localized, self-limited outbreaks that were relatively easy to control (World Health Organization, 2000). In the late 1990s, following El *Nino* rains, more wide-spread cholera outbreaks occurred. In subsequent years, outbreaks became an annual phenomenon (Bwire et al., 2013a,b). In 1997/1998 more than 90% of the districts in Uganda was affected by cholera outbreaks (Bwire et al., 2013b). The government of Uganda, working with development partners such as WHO and UNICEF, instituted control measures that included the promotion of access to safe water, sanitation and hygiene, treatment of patients, surveillance and health education. However, these strategies did not benefit communities equally, and cholera outbreaks continued to occur in Uganda nearly every year (Bwire et al., 2013a, 2017b).

Recent studies on cholera showed that cholera is endemic to Uganda and that outbreaks occurred frequently in specific districts located along the African Great Lakes in Uganda, the River Nile and on the international country borders (Alajo et al., 2006; Bwire et al., 2013b). The fishing villages in Uganda accounted for 58% of all reported cholera cases between 2011 and 2015 (Bwire et al., 2017b). There are two possible explanations for this observed distribution. One possible hypothesis is that the observed distribution of cholera along the major lakes in Uganda is that the lakes and the other surface water sources (rivers, wells, ponds, etc.) are environmental reservoirs for pathogenic *V. cholerae* responsible for cholera epidemics, as documented in Asia (Cabral, 2010; Wang et al., 2010; Lutz et al., 2013; Dalusi et al., 2015), and in epidemiological studies conducted in sub-Saharan African (Bompangue et al., 2008; Bompangue Nkoko et al., 2011; Kaddumukasa et al., 2012). It is possible that due to poverty, low level of awareness and inadequate access to safe water and sanitation, communities located along these surface water sources use water from these surface water sources without disinfection. Consequently, the communities get infected with pathogenic *V. cholerae* bacteria resulting in frequent cholera outbreaks.

A second hypothesis suggests that *V. cholerae* pathogens are introduced into these communities by humans, such as visitors, traders, relatives, refugees from the neighboring cholera endemic countries or communities with ongoing epidemics like the case of Haiti (Chin et al., 2011; Zarocostas, 2017). The introductions are thought to occur in a sustained manner due to endemic cholera in some of these neighboring countries, such as Democratic Republic of Congo (Shannon et al., 2015) and Kenya (Schaetti et al., 2013; Saidi et al., 2014). Irrespective of which of the two hypotheses is correct, available incidence data clearly show that the Great Lakes region has optimal conditions for transmission of *V. cholerae* resulting in repeated cholera outbreaks (Bompangue Nkoko et al., 2011; Bwire et al., 2017b).

In view of studies from Bangladesh and others on environmental reservoirs (Almagro-Moreno and Taylor, 2013; Righetto et al., 2015), the first hypothesis seems plausible. However, no comprehensive environmental study has been conducted on the various surface water sources in cholera-affected communities in Uganda to establish the presence of *V. cholerae*. Two studies attempted to document *V. cholerae* in Ugandan surface water sources, but these studies focused on few water sites on L. Victoria and the three Rift Valley lakes namely, L. George, Edward, and L. Albert (Kaddumukasa et al., 2012; Bagalwa et al., 2014). These earlier studies identified culturable *V. cholerae* but did not carry out agglutination tests to determine if they were epidemic serotypes O1 or O139. The aim of this study was to identify/detect epidemic *V. cholerae* and the potential for a reservoir for O1 and O139 in surface water sources (lakes, rivers, wells, ponds, a canal, and a drainage channel) located in cholera affected communities in African Great Lakes basin in Uganda.

## Materials and Methods

### Study Design and Area

**FIGURE 1:**
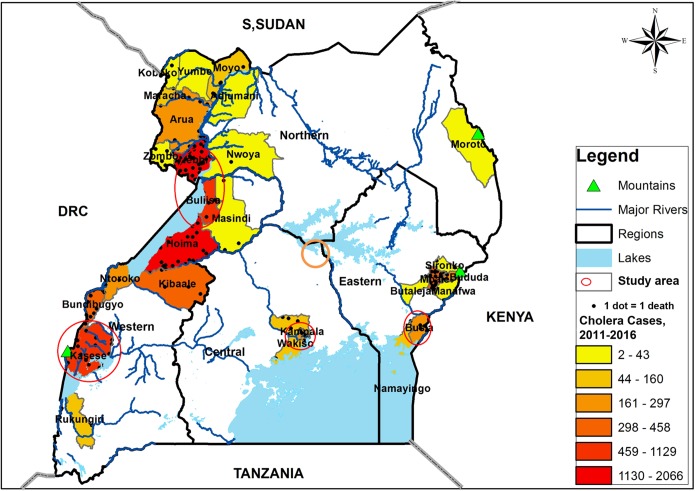
A map of Uganda showing the distribution of cholera cases for the period 2011–2015, the major surface water sources and the study area, February 2015–January 2016. The study area and water sampling sites were located in the communities with history of frequent cholera outbreaks.

This was a prospective study that was conducted for a period of 1 year (February 2015 to January 2016) in communities with recorded history of cholera outbreaks within the last 5–10 years, and were located in lake basins of the five African Great Lakes (Victoria, Albert, Edward, George, and Kyoga) in Uganda. Water from 28 sites was tested every month for the entire study period. Water samples were collected from lake water, rivers, wells, ponds, a canal, and drainage channel. The communities with ongoing or a previous record of cholera outbreaks and located in the lake basins of five African Great Lakes in Uganda were selected because they made up a majority of the annual reported cholera cases and deaths in Uganda (Bwire et al., 2017b). In order to identify the communities, we used aggregated district disease surveillance data on confirmed cholera outbreaks reported to the Ministry of Health Kampala for the period 2011–2015 (within the previous 5 year period), except for the sites on Lake Kyoga shores where we used the previous 10 years to select the district because the last outbreak occurred more than 5 years prior to the study. The study sites were in the districts of Kasese and Buliisa (western region), Busia (eastern region), Nebbi (northern region), Kampala, and Kayunga districts in central region of Uganda (**Figure 1**).

### Site Selection Procedure

The sites selected for water collection and testing were only those with surface water sources or points used by communities in areas with a history of cholera outbreaks within 5–10 years as described above. Surface water sources in the study area but not used by the communities for household purposes, such as L. Katwe in Kasese district, or harvested rainwater in water tanks were excluded from testing. We based the selection of the sites and water sources on the major lakes named above. A total of 28 points were identified, with support from community members and after direct field observations by the study team, to verify the use of water from these points for domestic purposes. Geographical positioning system (GPS) coordinates of these sites were captured and recorded to guide subsequent water sampling. On each of the Great Lakes, water samples were collected from two specific points that were often located in different districts, except for Kasese districts which had more than one lake within its district boundaries, namely, L. Edward and L. George. In addition to each selected lake site, a site on a river, well, pond, or canal in its neighborhood was selected for water testing following similar steps as those employed for the lake points (used by communities for household and domestic purposes, taking GPS coordinates). In addition an irrigation canal used by the local community for household purposes and a drainage channel that was discharging waste water into the lake next to the site of domestic water collection was tested.

### Water Sample Collection and Processing

Monthly water samples were collected at specific points with known GPS coordinates and at particular time of the day by laboratory technicians from the district hospitals. The laboratory technicians were members of the study team based at the district hospitals. A monthly schedule of all sites was included in the study protocol and was followed during implementation. Supervision of water collection was done by a senior laboratory technician on a monthly basis from Central Public Health Laboratory (CPHL) in Kampala. Three (3) liters of water were collected from each site and processed to test for the presence of *V. cholerae* according to previous documented method (Chakraborty et al., 2013; Debes et al., 2016b). Water samples were collected in a clean calibrated wide mouthed container from each point and assigned special sample identifier numbers corresponding to the district where the water site was located, water site, the date and time of sample collection. Water was collected and filtered immediately using fine folded sterile gauze designed to trap particles and bacteria attached to the particles as described by Spira and Ahmed (1981) and later by Chakraborty et al. (2013) and Debes et al. (2016b). Water filtration was done close to the site of water collection with entire filtration taking an average of 30–45 min. The gauze with its residual water was placed in a container with pre-packed double strength alkaline peptone water (APW) whose dilution changed to single strength (1% APW) after the insertion of the gauze and its contents. Thereafter, APW sample was packed in a cool box at room temperature (20–30°C) and taken to the district hospital where testing for the presence of *V. cholerae* was carried out the following day and after 24 h of incubation.

### Methods Used to Detect *V. cholerae* Bacteria in Water Samples

The three methods, namely enriched rapid test, culture, and multiplex polymerase chain reaction (PCR) were employed to test the water samples for the presence of epidemic *V. cholerae.* If the enriched rapid test was found positive, culture was to be carried out. The multiplex PCR method, employing specific primers, is able to detect DNA from *V. cholerae* and can differentiate *V. cholerae* O1, O139 and *V. cholerae* non O1/non O139 (Hoshino et al., 1998; Nandi et al., 2000; Huq et al., 2012; Mehrabadi et al., 2012)

#### Testing for *V. cholerae* O1 and O139 by Rapid Test and Culture Method

At the district laboratory the 1% APW samples, after a 24 h incubation period, were tested for the presence of epidemic *V. cholerae* O1, O139 or both using *Crystal VC* dipsticks (manufactured by Span Diagnostics Limited, Surat, India) according to previous methods (Debes et al., 2016b). If a sample yielded a positive result with the dipstick, a cotton swab was submerged in the enriched APW media and placed in a labeled transport container with Cary Blair transport media and shipped to the National Public Health Laboratory in Kampala within 24–48 h for culture. Furthermore, irrespective of whether the sample was positive with dipstick test, two drops of each 1% APW samples, after incubation for 24 h, were placed on pre-labeled Whatman filter paper (Merck Whatman^®^ protein saver cards 903 Protein saver card (US), pkg of 100 ea | Sigma-Aldrich, 2018) in duplicate and dried at room temperature (20–30°C) before safe packaging and shipment to Johns Hopkins University, Baltimore, MD, United States for multiplex PCR test to detect *V. cholerae* species. Special precautions were taken in handling the enriched APW samples to avoid environmental contamination and infecting the handlers (Huq et al., 2012).

#### Polymerase Chain Reaction Genotypying Procedure

In order to detect *V. cholerae* by PCR, DNA extractions from the filter papers were done using chelex-100 (Bio-Rad) according to previously described methods (Debes et al., 2016a). The type of *V. cholerae* present was identified through PCR amplification of specific genes namely: outer membrane protein *(OmpW), cholera enterotoxin sub-unit A (ctxA)*, and *rfb* genes according to previously described methods (Nandi et al., 2000). This method has the capacity to detect *V. cholerae* present in the sample and to differentiate nontoxigenic and toxigenic *V. cholerae*. Multiplex PCR targeted both an outer membrane protein gene, *OmpW*, which is a unique gene conserved in the *V. cholerae* genome, as well as cholera toxin A (*ctxA*) gene. We used the following PCR amplifier products 588, 301, and 308 bp to target *Omp*W, ctxA, and VCT genes, respectively. Any *V. cholerae* positive specimens, regardless of the presence of the toxin gene, were tested further with multiplex PCR to determine if they belonged to serogroup O1 or O139 using primers 192 and 449 bp, respectively (Hoshino et al., 1998). This multiplex PCR targets the *rfb* gene specific for the O1 and O139 serogroups. To ensure quality of PCR test, positive samples and those that were equivocal were re-run to give the final test results. The samples that were negative by both multiplex reactions were in addition tested for 16S rRNA gene by PCR to confirm bacterial genetic material presence in the extract (Hasan et al., 2011).

### Data Management

Each water sample was assigned a unique specimen identification (SID) number immediately after collection in the field. This number was used to identify the samples and accompanying form that went to laboratory with the samples. From the data on the paper forms, results were entered into a database using the Epiinfo^1^ seven statistical package. GIS coordinates were collected, cleaned, and stored in spreadsheet. Double entry and data cleaning was done to remove errors generated in the process data collection and entry. Analysis was done to get frequencies and percentages. Chi-squire test was used for comparison of surface water sources. We used GIS coordinates to create shapefiles that were combined with the location shapefiles to locate the position of the study sites on the map of Uganda. Shapefiles used to create the map of Uganda and administrative units were obtained from the Uganda Bureau of Statistics^2^. The maps were created using Arc View Geographical Information Software^3^ (Arc GIS).

### Ethical Consideration

Permission to conduct the study was obtained from Makerere University School of Public Health Institution Review Board (IRB 00011353) and Uganda National Council of Science and technology. Cholera data used to assist with selection of the surface water sources and study communities was from the Uganda Ministry of Health disease surveillance records. The surveillance data were aggregated reported cholera cases with no personal identifiers.

## Results

### General Description of the Study Sites

**FIGURE 2:**
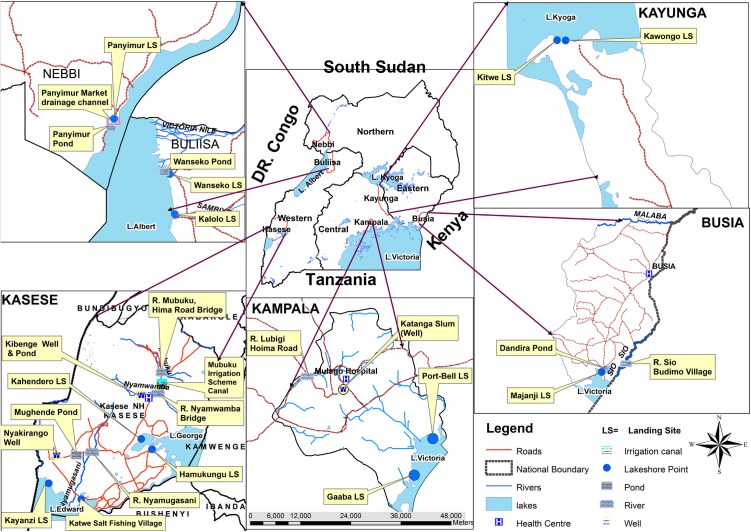
A map of Uganda showing selected *Vibrio cholerae* water sampling sites on the major surface water sources in Uganda, February 2015–January 2016.

A total of 322 water samples were collected from 28 sites during the 12 months of the study and tested for presence of *V. cholerae* O1, O139 and non O1/non O139. Forty-one percent (41%, 131/322) of samples tested were from major lakes; (26%, 84/322) from rivers; (18%, 57/322) from ponds; (11%, 34/322) from wells; (4%, 12/322) from irrigation canal; and 1% (4/12) from drainage channel located near a market in cholera endemic community, Nebbi district. The distribution of the study sites was as shown in **Figure 2**.

### Laboratory Test Results for *V. cholerae*

**FIGURE 3:**
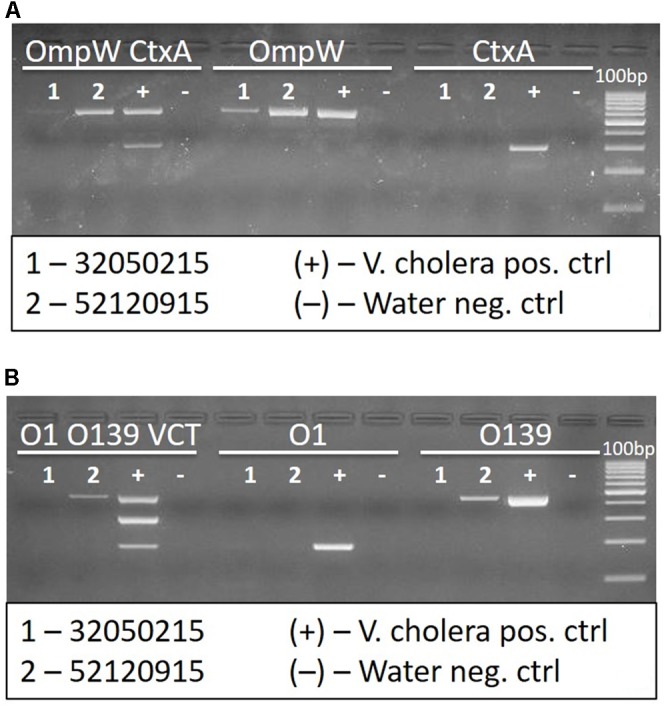
The PCR gel image showing *Vibrio cholerae* non-toxigenic non-O1 non-O139 sample (32050215) and non-toxigenic O139 sample (52120915) with controls. **(A)** *OmpW* *Ctx*A multiplex, *omp*W, and *ctx*A simplex reactions. **(B)** O1 O139 VCT multiplex, O1, and O139 simplex reactions. The bands for cholera toxin (VCT) and O1 are missing for the two samples. The band for O139 is seen but higher than usual (atypical position).

Of the 322 water samples tested with *Crystal VC* dipsticks, none were positive; thus, none were sent for culture. Two hundred and seventy-seven (277/322) samples were tested for *V. cholerae* using the PCR test. The other samples did not have sufficient DNA for analysis. Of these, 35 samples were positive for *V. cholerae* non O1/non O139, as determined by a positive band for *OmpW*. None of these were positive for cholera toxin as indicated by absence of a band for *ctxA*. Also, no toxigenic *V. cholerae* O1 or O139 band were observed by PCR test. PCR test identified two atypical *V. cholerae*, non-toxigenic O139 from a water sample collected in Kampala and Nebbi districts that needed further analysis by WGS tests. For these two samples the positive band on agarose gel picture was slightly higher than for the control even after two repeat PCR tests were done. The atypical PCR tests for the non O1 (SID 32050215) and non-toxigenic O139 (SID 520915) are shown in **Figure 3**.

**FIGURE 4:**
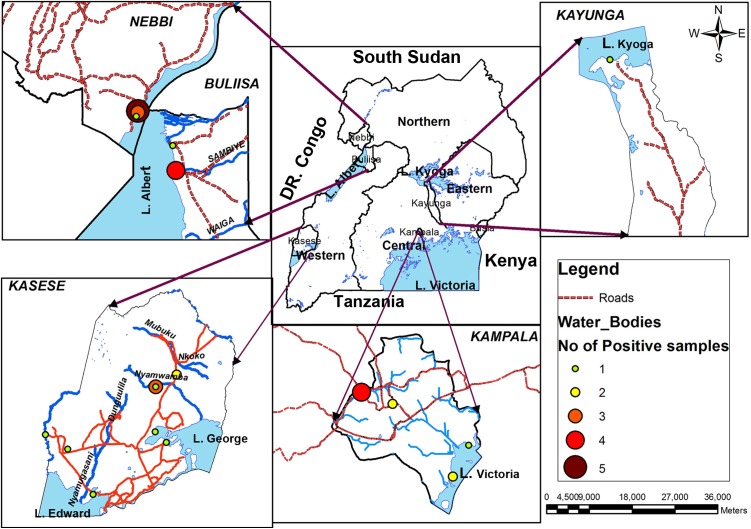
Geographical distribution of *Vibrio cholerae* non O1/non O139 in major surface water sources in Uganda, February 2015–January 2016. Lake Albert basin had the highest frequency while none were detected on eastern side of Lake Victoria, Busia district.

Further analysis of test results indicated that water samples collected in L. Albert basin had the highest number of positive test for non O1/non O139 *V. cholerae*. The drainage channel in Nebbi district on L. Albert had the highest percentage positive at 75%. However, only a few water samples were collected from these seasonal sites because later the channel dried up. The study sites and respective positives samples for non O1/non O139 were as shown in **Figure 4**.

### Distribution of *V. cholerae* Non O1/O139 by the Type of Water Body

A drainage channel had the highest percentage of positive samples while the ponds had the lowest. The number of positive water samples by the type of water sources and by the study districts is shown in **Tables 1**, **2**.

**Table 1:** Distribution of *Vibrio cholerae* non O1/non O139 in major surface water sources (lakes, rivers, wells, ponds, etc.) of Uganda, February 2015–January 2016.

Type of water	No. of sites	No. of sites with positive PCR test	No. of samples	Positive samples	Percent
Lakes	11	5	111	16	14%
Rivers	7	3	74	6	8%
Ponds	5	2	49	2	4%
Wells	3	3	29	6	21%
Irrigation canal	1	1	11	2	18%
Drainage channels	1	1	3	3	100%
Total samples from all sites	28	15	277	35	13%

**Table 2:** Distribution of *Vibrio cholerae* non O1/non O139 in major surface water sources by districts in Uganda during the study period, February 2015–January 2016.

District	Total sites	No of sites with positive samples	Total samples collected	No. positive samples	Percent
Kasese	13	8	133	11	8%
Buliisa	3	2	31	5	16%
Busia	3	0	32	0	0%
Kampala	4	4	40	9	23%
Kayunga	2	1	21	1	5%
Nebbi	3	3	20	9	45%
Total	28	18	277	35	13%

Water samples collected in Nebbi district had the highest number of positive test samples (45%, 9/20). No positive test water samples were recorded from L. Victoria basin, Busia district (lake, river, and pond water) during the 12 months study period.

### Monthly Distribution of *V. cholerae* Non O1/Non O139 in Surface Water Sources in Uganda

**FIGURE 5:**
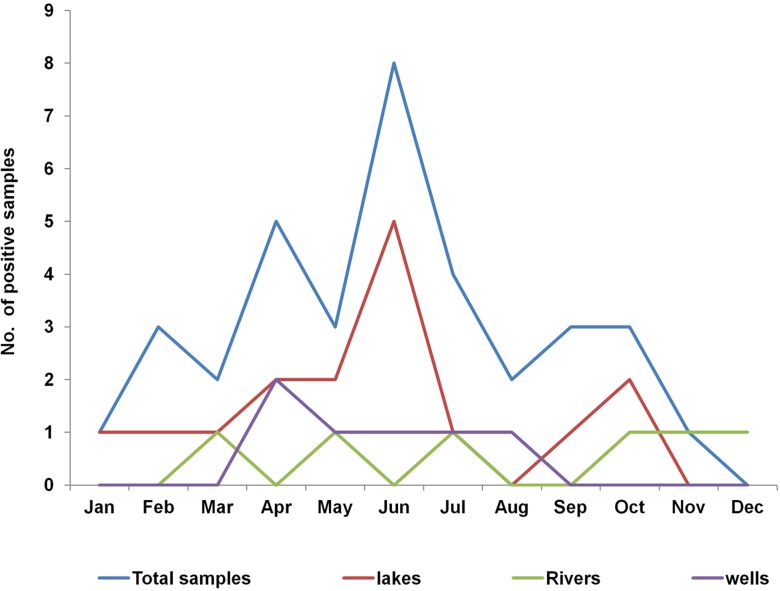
Monthly distribution of *Vibrio cholerae* non 01/non O139 in surface water sources in Uganda, February 2015–January 2016. There were two peak periods, the highest in April–June and a small one in October–November. The two peaks corresponded to the first (heavy rains) and second rainy (less heavy rains) seasons, respectively.

There was season variation of *V. cholerae* non O1/non O139 population in lakes and rivers with increase in positive tests during the rainy season with a first big peak in the month of March–June and another small peak in September–November. Monthly variation was as shown in **Figure 5**.

### Distribution of *V. cholerae* Non O1/Non 0139 in the Lake Water

**FIGURE 6:**
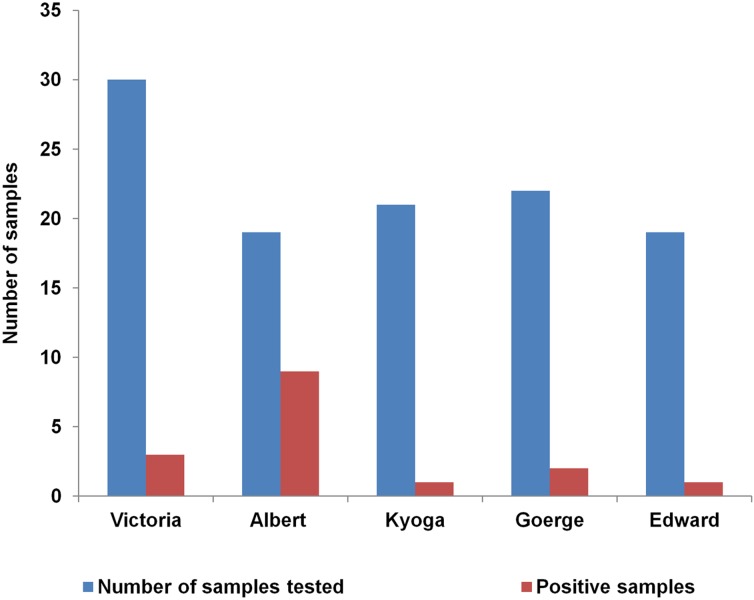
Distribution of *Vibrio cholerae* non O1/non O139 within the five African Great Lakes (Lakes: Victoria, Albert, Edward, George, and Kyoga) in Uganda, February 2015–January 2016. Lake Albert had more than double the number of positives isolated than the other lakes.

Further analysis of 111 dry spots from lakes was done. Lake Albert had the highest percentage of positive samples (47%, 9/19) which was 4.7 times higher than the next highest; L. Victoria which had (10%, 3/30) positive samples. The association between L. Albert and *V. cholerae* non O1/non O139 was statistically significant (*p* > 0.0001). Lake Kyoga had the least number of positive test samples. The proportions of *V. cholerae* non O1/O139 by each lake were as in **Figure 6**.

## Discussion

The findings of this study showed that the five African Great Lakes and other major surface water sources in Uganda were natural habitats for *V. cholerae* non O1/non O139 and atypical non-toxigenic *V. cholera*e O139 but no toxigenic (epidemic *V. cholerae* O1 or O139 were detected using our methods. *V. cholerae* non O1/non O139 were present frequently with the peak season in the months of March–July, corresponding to an increase during the rainy season. The favorable environmental conditions present during the rainy season may have been responsible for the observed peaking of non O1/non O139 in this study and these conditions could play a role in the spread *V. cholerae* O1 responsible for human cholera outbreaks once introduction has occurred. These favorable environmental and weather conditions during the rainy season may facilitate the seasonal pattern observed in human cholera outbreaks in the fishing villages in Uganda (Bwire et al., 2017b). Of the five lake basins studied, L. Albert basin was the region with the most frequent *V. cholerae* non O1/non O139 positive samples. Positive samples were found in different types of surface water sources, including rivers, wells, ponds, and drainage channels. Within the L. Victoria basin, *V. cholerae* non O1/non O139 were less common and they were not found in the eastern region of Uganda (Busia district) along the Uganda–Kenya border.

The absence of *V. cholerae* O1 suggests that although cholera outbreaks are common in the communities near the major lakes and rivers in Uganda, these lakes may not be the source of pathogens for repeated cholera outbreaks and are unlikely to be reservoirs for *V. cholerae* O1 as pointed out in earlier studies in sub-Saharan Africa (Bompangue Nkoko et al., 2011; Kaddumukasa et al., 2012) that used less definitive methods for pathogen detection. It seems more likely that the observed cholera outbreaks in Uganda could be due to other factors, such as human interaction from cholera endemic communities and outbreak affected areas leading to cross-border infections (Baluku et al., 2015; Bwire et al., 2016), traveling from cholera affected areas as happened in Juba, South Sudan (Ujjiga et al., 2015), since most of affected area were along busy international crossing points but also climatic factors could have contributed (Rebaudet et al., 2013).

More evidence in support of rejection of the hypothesis that the surface water sources (including the Great Lakes) in Uganda are reservoir for *V. cholerae* bacteria responsible for frequent cholera outbreaks is derived from molecular studies using PCR, multi-locus variable tandem repeat analysis (MLVA) and WGS of *V. cholerae* isolates collected during cholera outbreaks in Uganda for the period 2014–2016 and those responsible for cholera outbreaks in Africa over several years (Bwire et al., 2018) and in South Sudan and Uganda (Abubakar et al., 2018). These studies showed close genetic relatedness and spread of *V. cholerae* species within Uganda; spread into Uganda across international boundary from the neighboring countries in the region namely Democratic Republic of Congo, South Sudan, and Tanzania and spread out of Uganda into Tanzania and South Sudan and vice versa.

Although the lakes may not be reservoirs, they may still facilitate this cross-border transmission by providing a permissive environment for *V. cholerae* spread or by accelerating the number of people crossing the border since few movement barriers exist along the international borders in the lakes. Such cross-border outbreaks present a major challenge to cholera control (Bwire et al., 2016). The cholera-affected communities along the country borders have inadequate access to safe water, sanitation (Baluku et al., 2015), hygiene and high level of poverty, and these factors may be important contributors, just as they contribute to the outbreaks in informal settlement (slum areas) in urban settings (Sur et al., 2005). Likewise, sustained poor sanitation which is common in these communities possibly results in frequent contamination of water sources with fecal matter or sewage (Okui, 2013; Kwesiga et al., 2017; Oguttu et al., 2017) which then facilitate and contribute to persistent cholera epidemics in similar manner to cholera epidemic in London before John Snow identified the problem (Snow, 1855).

The fact that these outbreaks occur in communities along the lakes with poor sanitation but with frequent cross-border movements (Bwire et al., 2017a), but rarely in lake communities with poor sanitation but away from border movements is further evidence to reject the hypothesis that the Great Lakes in Uganda are reservoirs for epidemic *V. cholerae* pathogens. In fact, studies on cholera in the fishing villages (Bwire et al., 2017b) did not document outbreaks along the lakes George and Kyoga which are in the interior of Uganda but have no cross-border movements. Similarly, island districts such as Kalangala and Buvuma districts that are located in L. Victoria with inadequate access to safe water, with fishing community that have localized activities were also not cholera endemic districts.

The conclusion that the major surface water sources in Uganda may not be the reservoir for epidemic *V. cholerae* is also supported by a study on epidemiology of cholera in the fishing villages. The communities in the fishing villages were responsible for majority (58%) of the infections that occurred in Uganda in the period 2011–2015 (Bwire et al., 2017b). The highest burden among the fishing villages was in the communities in the districts along the international borders on these lakes and rivers. However, similar fishing villages on the same lakes but located in the interior of Uganda away from the international boarders in districts such as Mukono, Jinja, Buvuma, and Masaka on L Victoria and Amolatar, Nakasongola and Apac along the Nile river with similar conditions did not report cholera outbreaks (Bwire et al., 2017b).

Finally, although the lakes were not reservoir for epidemic *V. cholerae* the presence of *V. cholerae* non O1/non O139 that are known to cause localized outbreaks in some communities (Bagchi et al., 1993) and diarrhea in travelers (Bhattacharya et al., 1992) and other disease conditions such as septicemia, enteritis, bacteremia among other infections (Chen et al., 2015; Deshayes et al., 2015; Kaki et al., 2017) is important for public health. Could it be that some of the localized outbreaks occurring in these communities are due to these pathogens? Also, *V. cholerae* are known to acquire pathogenicity factors and virulence regulators encoded in mobile genetic elements from the environment and lysogenic phages during biotic and abiotic pressures (Sakib et al., 2018). Since *V. cholerae* were naturally present in these water it is possible for this mechanism to play a role in emergence of virulence straits. Therefore, additional studies would be required to determine the role that *V. cholerae* non O1/non O139 and atypical non toxigenic *V. cholerae* O139 may play in these other diseases. Similarly, because the distribution of *V. cholerae* non O1/non O139 was not uniform across the test sites, physicochemical characteristics of these surface water sources should be studied to guide establishment of surveillance system to monitor and prevent these diseases (Centre for Disease Control and Prevention [CDC], 2012).

### Strengths of the Study

The strength of this study lies in the following facts: First, the investigators purposively selected sites in communities with a high cholera burden, as guided by epidemiological data from the Ministry of Health Uganda. This purposive selection of water sites would result in detection of toxigenic (epidemic) *V. cholerae* O1 or O139 if it were present. Second, the fact that the test methods were frequently able to detect *V. cholerae* non O1/non O139 and atypical non-toxigenic O139 demonstrates that these methods were suitable for *V. cholerae* (Debes et al., 2016a). Third, detection methods that were used were also found efficient in other studies for detection of Vibrios (Chakraborty et al., 2013; Debes et al., 2016b) and finally, the conclusion of this study, of no evidence to support the surface water sources serving as reservoir for epidemic *V. cholerae* is also backed by both epidemiologic and molecular studies on spread of cholera and genetic relatedness of *V. cholerae* bacteria responsible for cholera outbreaks in Uganda and sub-Saharan Africa (Abubakar et al., 2018; Bwire et al., 2018).

### Weakness and Limitations of the Study

Some limitations of this study included the following. First, lack of an efficient methods to detect all the forms of *V. cholerae* since some Vibrios exist either as surface-associated or as free-living lifestyle in aquatic environments (Dang and Lovell, 2016). Also, *V. cholerae* may colonize the surfaces of different objects such as phytoplankton, zooplankton, aggregates, sediments, rocks, and some *V. cholerae* may enter the viable-but-nonculturable (VBNC) state (Huq and Colwell, 1994; Dang and Lovell, 2016) and also associate with fowl, fish, and other sea dwellers (Almagro-Moreno and Taylor, 2013). However, since the study used multiplex PCR to detect the DNA from *V. cholerae*, it is possible to have detected such VBNC if it were present (Liu et al., 2014), but the methods were not designed for this purpose. Second, the study involved testing of water samples from surface water sources and components therein only, it is possible that the reservoirs could be in other organisms such as fish, snails, etc. (Senderovich et al., 2010) which communities use for various activities including feeding but were not considered in this study. Therefore, further studies using appropriate method and targeting ecosystem not covered by our study should be conducted to provide more information on *V. cholerae* in surface water sources. Finally, this study was conducted for a period of 1 year; possibly the results might have been different if it were to continue for more years; however, we believe that this time was adequate for us to establish the presence of *V. cholerae* O1 or O139 in a cholera endemic locations (Bwire et al., 2017a).

## Conclusion

This study found no evidence that the African Great Lakes and other surface water sources in Uganda were reservoirs for *V. cholerae* O1 or O139 that cause cholera epidemics in humans. However, these lakes were natural habitats for *V. cholerae* non O1 and non O139 and also provided conducive conditions for the spread and propagation of the epidemic *V. cholerae* once introduction had occurred in an area. Most importantly, this study provided useful information that could be used to strengthen interventions for prevention, control, and elimination of cholera in Uganda and neighboring counties in East Africa. More studies are needed to ascertain the environmental conditions in the surface water sources that facilitated the existence *V. cholerae* non O1/non O139 and non-toxigenic *V. cholerae* O139. *V. cholerae* non O1/non O139 infections disease surveillance system should be established in Uganda and East African region. In addition WGS of the atypical *V. cholerae* identified by multiplex PCR should be conducted to provide better understanding of the detected bacteria/organisms.

## Author Contributions

GB, AD, DS, and CO have conceived and designed the experiments. GB, AD, AK, HK, and JV have performed the experiments. GB, MR, HK, WAB, JV, and AD have analyzed the data. GB, CO, DS, TO, AK, and MR have supervised the implementation. GB and CO have wrote the first draft of the manuscript. GB, DS, AD, JV, TO, AK, WAB, MR, HK, and AWB have provided critical input into the first draft of the manuscript. GB, DS, AD, CO, AK, JV, MR, HK, TO, WAB, and AWB have contributed to the final manuscript.

## Conflict of Interest Statement

The authors declare that the research was conducted in the absence of any commercial or financial relationships that could be construed as a potential conflict of interest.

## Footnotes

1 https://www.cdc.gov/epiinfo/pc.html

2 http://www.ubos.org/statistical-activities/

3 https://www.arcgis.com
